# Supplementary material for: Enhancing in vitro ruminal digestibility of oil palm empty fruit bunch by biological pre-treatment with Ganoderma lucidum fungal culture
Source: PLoS One. 2021 Sep 30;16(9):e0258065. doi: 10.1371/journal.pone.0258065 (PMC8483372; doi:10.1371/journal.pone.0258065)
Supplement: S1 Table — (DOCX) [file pone.0258065.s001.docx]

**S1 Table. Changes in fibre composition and dry matter of OPEFB pre-treated with *G. lucidum* across treatment period were fitted to linear model.**

| **Variable** | **Pre-treatment period (week)** | | | | | | | **R^2^ value of linear regression** |
| --- | --- | --- | --- | --- | --- | --- | --- | --- |
|  | **0** | **2** | **4** | **6** | **8** | **10** | **12** |  |
| **Neutral detergent fibre (g kg^-1^ DM)** | 251.12 ±5.61a | 246.35 ±7.05ab | 241.90 ±2.62ab | 223.88 ±2.42cd | 220.93 ±1.97cd | 211.93 ±1.55de | 202.88 ±6.20e | 0.9728 |
| **Acid detergent fibre (g kg^-1^ DM)** | 196.71 ±2.90a | 189.92 ±5.92abc | 190.56 ±3.10ab | 177.23 ±1.18cd | 178.36 ±0.70bcd | 169.42 ±1.69de | 162.99 ±6.77e | 0.9487 |
| **Lignin**  **(g kg^-1^ DM)** | 35.77 ±0.76a | 29.26 ±0.90b | 28.33 ±0.56bc | 25.20 ±0.17bcd | 24.19 ±1.27cd | 23.28 ±0.76d | 21.11 ±0.90d | 0.9016 |
| **Cellulose**  **(g kg^-1^ DM)** | 78.28 ±1.89abc | 81.69 ±0.96a | 79.65 ±1.72ab | 75.92 ±1.04bc | 73.77 ±1.89c | 67.59 ±0.90d | 62.24 ±0.79e | 0.8255 |
| **Hemicellulose**  **(g kg^-1^ DM)** | 54.41 ±2.96a | 56.44 ±1.75a | 51.36 ±3.04ab | 46.65 ±1.41bc | 42.57 ±1.58c | 42.51 ±1.27c | 39.89 ±0.96c | 0.9185 |
| **Dry matter**  **(g kg^-1^ fresh substrate)** | 79.47 ±0.87cd | 76.54 ±1.49cd | 82.03 ±1.38c | 77.07 ±0.96cd | 94.63 ±2.06b | 115.44 ±2.28a | 111.49 ±1.47a | 0.7541 |

Data are expressed as the means ± standard errors. Means with the same letter in each row indicates not significantly different (*p* ≥ 0.05, Duncan’s multiple range test).
