# Supplementary material for: Enhancing in vitro ruminal digestibility of oil palm empty fruit bunch by biological pre-treatment with Ganoderma lucidum fungal culture
Source: PLoS One. 2021 Sep 30;16(9):e0258065. doi: 10.1371/journal.pone.0258065 (PMC8483372; doi:10.1371/journal.pone.0258065)
Supplement: S2 Table — (DOCX) [file pone.0258065.s002.docx]

**S2 Table. In vitro gas production (mL 200 mg^-1^ dry matter) of OPEFB pre-treated with *G. lucidum* across incubation period.**

| **In vitro incubation time (hour)** | **Pre-treatment period (week)** | | | | | | | ***p* value** |
| --- | --- | --- | --- | --- | --- | --- | --- | --- |
|  | **0** | **2** | **4** | **6** | **8** | **10** | **12** |  |
| **2** | 4.70 ±0.83c | 3.29 ±0.31de | 2.53 ±0.18e | 4.20 ±0.31cd | 6.39 ±0.12b | 6.70 ±0.30b | 7.94 ±0.18a | <0.0001 |
| **4** | 8.67 ±1.46c | 6.33 ±0.54de | 4.93 ±0.35e | 8.18 ±0.59cd | 12.19 ±0.26b | 12.64 ±0.55b | 15.03 ±0.37a | <0.0001 |
| **6** | 12.57 ±1.95c | 9.15 ±0.71de | 7.21 ±0.52e | 11.94 ±0.83cd | 17.44 ±0.40b | 17.89 ±0.74b | 21.35 ±0.54a | <0.0001 |
| **8** | 15.85 ±2.29c | 11.77 ±0.84d | 9.37 ±0.69d | 15.51 ±1.03c | 22.20 ±0.56b | 22.54 ±0.89b | 27.00 ±0.72a | <0.0001 |
| **10** | 18.78 ±2.53c | 14.20 ±0.91d | 11.41 ±0.85d | 18.90 ±1.20c | 26.51 ±0.73b | 26.66 ±1.00b | 32.03 ±0.88a | <0.0001 |
| **12** | 21.38 ±2.68c | 16.44 ±0.95d | 13.36 ±1.01d | 22.10 ±1.35c | 30.43 ±0.90b | 30.31 ±1.09b | 36.52 ±1.04a | <0.0001 |
| **24** | 32.01 ±2.50d | 26.96 ±0.73de | 23.08 ±1.92e | 38.12 ±1.77c | 47.32 ±1.90b | 44.92 ±1.24b | 54.96 ±1.84a | <0.0001 |
| **36** | 37.71 ±1.70d | 33.79 ±0.80de | 30.16 ±2.72e | 49.76 ±1.66c | 56.71 ±2.76b | 51.97 ±1.17bc | 64.27 ±2.39a | <0.0001 |
| **48** | 40.78 ±1.23c | 38.29 ±1.51c | 35.32 ±3.40c | 58.24 ±1.28b | 61.95 ±3.40b | 55.38 ±1.09b | 68.97 ±2.74a | <0.0001 |
| **72** | 43.55 ±1.75c | 43.33 ±2.86c | 41.83 ±4.46c | 68.97 ±0.33a | 66.51 ±4.15ab | 57.83 ±0.10b | 72.56 ±3.08a | <0.0001 |

Data are expressed as the means ± standard errors. Means with the same letter in each row indicates not significantly different (*p* ≥ 0.05, Duncan’s multiple range test).
