# Supplementary material for: Enhancing in vitro ruminal digestibility of oil palm empty fruit bunch by biological pre-treatment with Ganoderma lucidum fungal culture
Source: PLoS One. 2021 Sep 30;16(9):e0258065. doi: 10.1371/journal.pone.0258065 (PMC8483372; doi:10.1371/journal.pone.0258065)
Supplement: S3 Table — (DOCX) [file pone.0258065.s003.docx]

**S3 Table. In vitro dry matter and organic matter digestibility of OPEFB pre-treated with *G. lucidum* at 48 hours of in vitro gas production.**

| Pre-treatment period (week) | In vitro digestibility (%) | |
| --- | --- | --- |
|  | Dry matter digestibility | Organic matter digestibility |
| **0** | 37.83 ±1.01c | 35.55 ±2.50c |
| **2** | 38.75 ±0.75c | 37.00 ±2.00c |
| **4** | 36.00 ±2.50c | 35.50 ±1.50c |
| **6** | 58.75 ±0.25b | 54.20 ±4.80b |
| **8** | 65.00 ±1.53b | 60.31 ±2.22ab |
| **10** | 59.33 ±3.18b | 54.25 ±0.75b |
| **12** | 77.20 ±1.51a | 69.78 ±4.58a |
| ***p* value** | <0.0001 | 0.0002 |

Data are expressed as the means ± standard errors. Means with the same letter in each column indicates not significantly different (*p* ≥ 0.05, Duncan’s multiple range test).
