# Supplementary material for: Enhancing in vitro ruminal digestibility of oil palm empty fruit bunch by biological pre-treatment with Ganoderma lucidum fungal culture
Source: PLoS One. 2021 Sep 30;16(9):e0258065. doi: 10.1371/journal.pone.0258065 (PMC8483372; doi:10.1371/journal.pone.0258065)
Supplement: Attachment — Submitted filename: Response to Reviewers.pdf [file pone.0258065.s004.pdf]

RESPONSE TO REVIEWERS

Date: June 17, 2021

Dear Sir,

The authors would like to thank the reviewers for their specific and helpful comments and suggestions. Please find enclosed the revised manuscript with marked changes (Revised Manuscript with Track Changes) and unmarked (Manuscript).

Manuscript title	:	Enhancing in vitro ruminal digestibility of oil palm empty fruit bunch by biological pre-treatment with *Ganoderma lucidum* fungal culture
Type of article	:	Research article
Manuscript ID	:	PONE-D-21-15493

The authors response to the reviewers are as in APPENDIX 1.

We hope that our revised manuscript is considerable for publication in PLOS ONE.

Thank you.

Your sincerely,

Rakib

Corresponding Author

APPENDIX 1

Journals Requirements		
**No.**	**Issue/comment/suggestion by the reviewer**	**Response by the authors**
1.	Please ensure that your manuscript meets PLOS ONE's style requirements, including those for file naming.	Contribution statements were added on the title page. The authors also checked throughout the manuscript to ensure the style and formatting. File naming followed the guide given.

Reviewer 1		
**No.**	**Issue/comment/suggestion by the reviewer**	**Response by the authors**
2.	The work was not well set for objectives to be investigated.	As stated in the manuscript, the objectives of the study were to evaluate the changes in fibre composition and in vitro rumen digestibility of OPEFB pre-treated with *Ganoderma lucidum* fungal culture across different incubation period. We believed that all experiments were well set to achieve the objectives, as stated by Reviewer 2. The fibre composition was recorded for every 2 weeks interval for a period of 12 weeks of incubation, and uninoculated OPEFB served as control. Then, the OPEFB pre-treated for different duration was further evaluated for in vitro rumen gas production and digestibility. Treated and untreated samples of OPEFB were also analyzed under SEM to observe physical changes. Therefore, we disagree to the comment by Reviewer 1.
3.	Many research works in this area has been reported but the authors have not made sufficient reviews with relevant data.	Many relevant references that related to the use of crop residue, and biological/fungal treatments of crop residues were reviewed and cited. Among of the references are No. 10, 12, 13, 14, 15, 16, 27, 33, and 42.
4.	No new innovations have been found and reported under this experiment.	There was no published article or data found on the use of *Ganoderma lucidum* to biologically delignify OPEFB, and improve its in vitro ruminal digestibility. Other studies reported the use of different

		organic materials as the forage, and different fungi species for pre-treatment. Therefore, we agreed with Reviewer 2, and disagreed with Reviewer 1.
5.	Both the Abstract and Conclusion were not well wrapped up using the significant findings under this experiment.	The abstract was modified to include the key findings of the study. The conclusion was not modified because the authors believed that there is no need to mention the results data in the conclusion section, as this will cause redundancy of information.
6.	In particular, comparison of the treated OPEFB after various periods should have been stated comparatively among the treatments.	The changes of fiber composition, and in vitro rumen digestibility of the OPEFB among the treatments (across pre-treatment period) were already stated clearly in the results of the study. Thus, no further change was made.
7.	The authors did not show any suggestions or recommendations to further implement the treated OPEFB in the possible feeding interventions.	The limitations, recommendations for future research, and potential applications of the treated OPEFB were stated in the final paragraph of the results and discussion section (L362 - L373).

**Reviewer 2**		
**No.**	**Issue/comment/suggestion by the reviewer**	**Response by the authors**
8.	The study investigated the enhancing of the nutritive value of oil palm empty fruit bunch after a fungal culture treatment. This is a comprehensive and very interesting study for increasing the nutritive value after reducing the lignocellulose content by fungal degradation. The study was well-conducted with reasonable replicates.	The authors would like to appreciate the merits given to the study and manuscript.
9.	Because Reishi is used in traditional medicine in the East, its price is high. Would it be profitable to use it to improve the nutritional value of fruit and vegetable by-products? that is, is the process very expensive? Well, when a fruit and vegetable by-product is used, it is to replace a more expensive food. And if the treatment of the by-product with Reishi is expensive, then the cost of the ration will not be lowered. have you evaluated these aspects? Could Reishi affect palatability?	The authors understood the concern raised by the reviewer. Firstly, the *G. lucidum* or Reishi used in traditional medicine is the basidiocarp (fruiting body). However, the present study used mycelium culture which is easier to culture and multiply on carbon rich organic materials. Unfortunately, the cost-effective aspect was not evaluated in present study, but the authors believe that the use of mycelial culture of *G. lucidum* will

		not affect on the cost much as they can easily proliferate. The cost-effective aspect can be evaluated in a study after the treated OPEFB is mixed with other ingredients to produce a balanced diet for ruminants, as stated in L372.
10.	L10-L13-L153-L155-L157 …. Universiti? Check English language, please.	The authors are aware of the spelling 'Universiti'. Universiti Malaysia Sabah, and Universiti Putra Malaysia are proper nouns without English name. Changing the university name to University of Malaysia Sabah, and University of Putra Malaysia is inappropriate. Therefore, no changes were made.
11.	L77 new paragraph, so tabulate.	Tabulated.
12.	L88 After Ganoderna lucidum add (G. Lucidum).	The authors think that adding an abbreviated scientific name after its full name is unnecessary. No change was made in the manuscript.
13.	L170 NEWAY is any Technology, Company, please clarify.	NEWAY is a computer program developed by The Rowett Institute, The University of Aberdeen. The terms NEWAY also commonly used. No change was made in the manuscript.
14.	L185 Do you use DMRT abbreviation though the manuscript? If not, you do not need it.	The abbreviation was used once, and its full name was stated throughout the manuscript. Thus, the abbreviation 'DMRT' was removed from the manuscript as suggested by the reviewer.
15.	L189 SAS is Statistical Analysis System.	Changed to 'Statistical Analysis System'.
16.	L198 I am sorry I could not find what is viz.?	The word 'viz.' is an abbreviation of the Latin phrase videre licet, synonym for the word 'namely', 'which is', or 'as follows'. The word 'viz.' in the manuscript was changed to 'namely', which is a more common word.
17.	L303-312 correlation is R or r? Please revise the authors guidelines.	The authors have check through few published articles in PLOS ONE, and found that the symbol of correlation coefficient are presented as either 'R' or 'r'. Therefore, the authors decided to maintain the symbol 'R' throughout the manuscript.

18.	As I have commented previously, the study is very interesting and the description of the in vitro techniques for the production of gas and the degradability of the dry matter are adequate. But since the main objective is to use this waste byproduct in the feeding of ruminant animals, I miss the determination of some parameters and indices related to ruminal fermentation. I believe that to continue with the publication of this manuscript it should be essential to incorporate at least one table with the pH, ammoniacal nitrogen, total volatile fatty acids, and some individual VFA such us acetate, butyrate, propionate, isobutyrate, valerate and isovalerate. And ideally, it would be possible to have some odd-chain volatile fatty acid. But I think that at least these basic ones that I have indicated must be included. It is probable that the authors have already analyzed them and can incorporate them.	The author agreed that it will be good if the rumen fermentation characteristics data as stated by the reviewer is included in this manuscript. Unfortunately, such data was not recorded during the study, and the authors are unable to provide the data due to unavailability of samples to redo the in vitro experiment. The authors stated in L369 and L370, that the effect of finalized feed formulation (balanced ration) on rumen quality or fermentation characteristics should be evaluated in future study. The present study focused on improving the digestibility of OPEFB. The authors also believed that we provided sufficient data to support that the treatment of *G. lucidum* on OPEFB was able to reduce the fibre composition (especially lignin), and enhanced the in vitro rumen digestibility.
19.	The results and the technology are very interesting. English grammar and expression should be improved.	Thanks for the merits given by the reviewer on the results and technology of this study. Few language issues as in No. 10, 12, 14, 15, 16 and 17 were clarified and modified (if applicable).
