# Supplementary material for: Enhancing in vitro ruminal digestibility of oil palm empty fruit bunch by biological pre-treatment with Ganoderma lucidum fungal culture
Source: PLoS One. 2021 Sep 30;16(9):e0258065. doi: 10.1371/journal.pone.0258065 (PMC8483372; doi:10.1371/journal.pone.0258065)
Supplement: Attachment — Submitted filename: Response to Reviewers.pdf [file pone.0258065.s005.pdf]

RESPONSE TO REVIEWERS

Date: September 7, 2021

Dear Sir,

The authors would like to thank the reviewers for their specific and helpful comments and suggestions. Please find enclosed the revised manuscript with marked changes (Revised Manuscript with Track Changes) and unmarked (Manuscript).

Manuscript title	:	Enhancing in vitro ruminal digestibility of oil palm empty fruit bunch by biological pre-treatment with *Ganoderma lucidum* fungal culture
Type of article	:	Research article
Manuscript ID	:	PONE-D-21-15493R1

The authors response to the reviewers are as in APPENDIX 1.

We hope that our revised manuscript is considerable for publication in PLOS ONE.

Thank you.

Your sincerely,

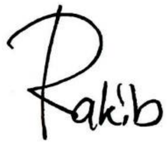

Corresponding Author

APPENDIX 1

Journals Requirements		
**No.**	**Issue/comment/suggestion by the reviewer**	**Response by the authors**
1.	Please review your reference list to ensure that it is complete and correct. If you have cited papers that have been retracted, please include the rationale for doing so in the manuscript text, or remove these references and replace them with relevant current references. Any changes to the reference list should be mentioned in the rebuttal letter that accompanies your revised manuscript. If you need to cite a retracted article, indicate the article's retracted status in the References list and also include a citation and full reference for the retraction notice.	The citations and reference list were cross-checked, and ensured complete and correct.<br><br>Reference No. 23 was updated from "Zahari MJI, Mohd Jahi N, Hanisah MN, Ahmad I, Baharum A, Mat Lazim A, et al. Enhanced performance of cellulose from palm oil empty fruit bunch (EFB) via acetylation and silylation. Preprints. 2018: 2018070314. doi: 10.20944/preprints201807.0314.v1." to "Mohammad IN, Ongkudon CM, Misson M. Physicochemical properties and lignin degradation of thermal-pretreated oil palm empty fruit bunch. Energies. 2020;13: 5966. doi: 10.3390/en13225966.". This due to the initial reference (Zahari et al. 2018) found as preprints, and no published article was found. This study used similar procedure according to Mohammad et al. (2020).

Reviewer #1		
**No.**	**Issue/comment/suggestion by the reviewer**	**Response by the authors**
2.	Although, the authors have addressed partly to the issues raised, some additional clarifications and modifications are still required:<br><br>Line; 370-373, based on the improved nutritional value of the OPEFB by *G. lucidum*, the authors need to address the improvement and express the lacking parts ..and propose the potential improvement. It would be beneficial to refer to previous workers-References.	The lacking of the *G. lucidum*-treated OPEFB was stated, where it contain low amount of crude protein, lipids, vitamins, and minerals. Therefore, it is necessary to incorporate the treated OPEFB with other nutrients as supplements for formulation of a balanced ration. Two additional references were added ([49,50]).
3.	Conclusions: It looks as if, the authors still discussing, rather than summarizing the significant findings under this experiment, in particular to the improvement rendered *by G. lucidum*, to what extent?	The extent of changes in the *G. lucidum* treated OPEFB were specified in the conclusion section, the conclusion summarized the major findings, and answered the objectives of the study.

Reviewer #2		
**No.**	**Issue/comment/suggestion by the reviewer**	**Response by the authors**
4.	The manuscript is now ready for publication. The authors have responded to all the questions that were raised and have completed and improved the document.	The authors would like to thank Reviewer #2 for his/her kind contribution in reviewing this manuscript.

Other issue revised by the authors		
	**Issue**	**Modification**
5.	The rate of inoculum used to inoculate the OPEFB.	Previously, the rate stated was based on the number of mycelia plugs (5 mm) per 500 g OPEFB. The rate now is expressed as mg kg^{-1}.
